# Successful Treatment of Median Nerve Mononeuropathy Occurring After Brachial Arterial Line Placement for a Prolonged Prone Spine Surgery With Ultrasound-Guided Peripheral Nerve Stimulation: A Case Report

**DOI:** 10.7759/cureus.95398

**Published:** 2025-10-25

**Authors:** Varun Channagiri

**Affiliations:** 1 Anesthesiology, Moffitt Cancer Center, Tampa, USA

**Keywords:** brachial arterial line, neuropathy of median nerve, peripheral nerve stimulation, postoperative neuropathy, prone spine surgery, ultrasound-guided

## Abstract

Median nerve injury after brachial arterial access is uncommon but clinically meaningful, typically related to direct needle trauma, hematoma, pseudoaneurysm, or ischemia from arterial compromise. We report a case of a 66-year-old man with persistent neuropathic pain and sensory loss in the thumb to middle fingers, with symptom onset after a 12-hour prone spine surgery during which a brachial arterial line was placed. Symptoms persisted for more than one year despite conservative therapy. Electrodiagnostic testing demonstrated a chronic focal mononeuropathy of the median nerve with superimposed sensory polyneuropathy. Under ultrasound guidance, a percutaneous 60-day peripheral nerve stimulation (PNS) lead (SPRINT^®^, SPR Therapeutics, Cleveland, OH) was placed adjacent to the proximal median nerve. At two-month follow-up, the patient reported greater than 50% pain reduction, improved dexterity, and increased participation in therapy, with continued benefit on neuropathic medication and topical analgesic. This case highlights a delayed presentation of median neuropathy likely related to prior brachial arterial cannulation and supports ultrasound-guided PNS as a feasible, minimally invasive option when conservative measures fail. Recognition of this iatrogenic mechanism is important because timely evaluation and targeted neuromodulation can improve function and quality of life.

## Introduction

Median nerve mononeuropathy most commonly reflects distal compression at the wrist; however, proximal injury can occur after vascular access in the antecubital fossa, where the brachial artery and median nerve lie in close proximity. Reported median nerve complications after brachial artery catheterization are uncommon but may be underrecognized and can lead to persistent functional deficits if not recognized and addressed in a timely fashion [1]. Mechanisms include direct needle trauma, hematoma or pseudoaneurysm causing compression, and ischemic injury; the risk may be higher with axillary or brachial access than with more superficial radial access [2]. Prolonged prone positioning and difficult hemostasis may further predispose to nerve injury. We describe a delayed median neuropathy after brachial arterial line placement for a prolonged spine surgery, successfully managed with ultrasound-guided percutaneous 60-day peripheral nerve stimulation (PNS).

## Case presentation

A 66-year-old man presented with chronic neuropathic pain and numbness in the thumb, index, and middle fingers of the left hand beginning after a prolonged (approximately 12-hour) prone spine procedure during which a brachial arterial line was placed for monitoring. Symptoms persisted for more than one year and were refractory to splinting, activity modification, physical and occupational therapy, and neuropathic agents.

**Figure 1:**
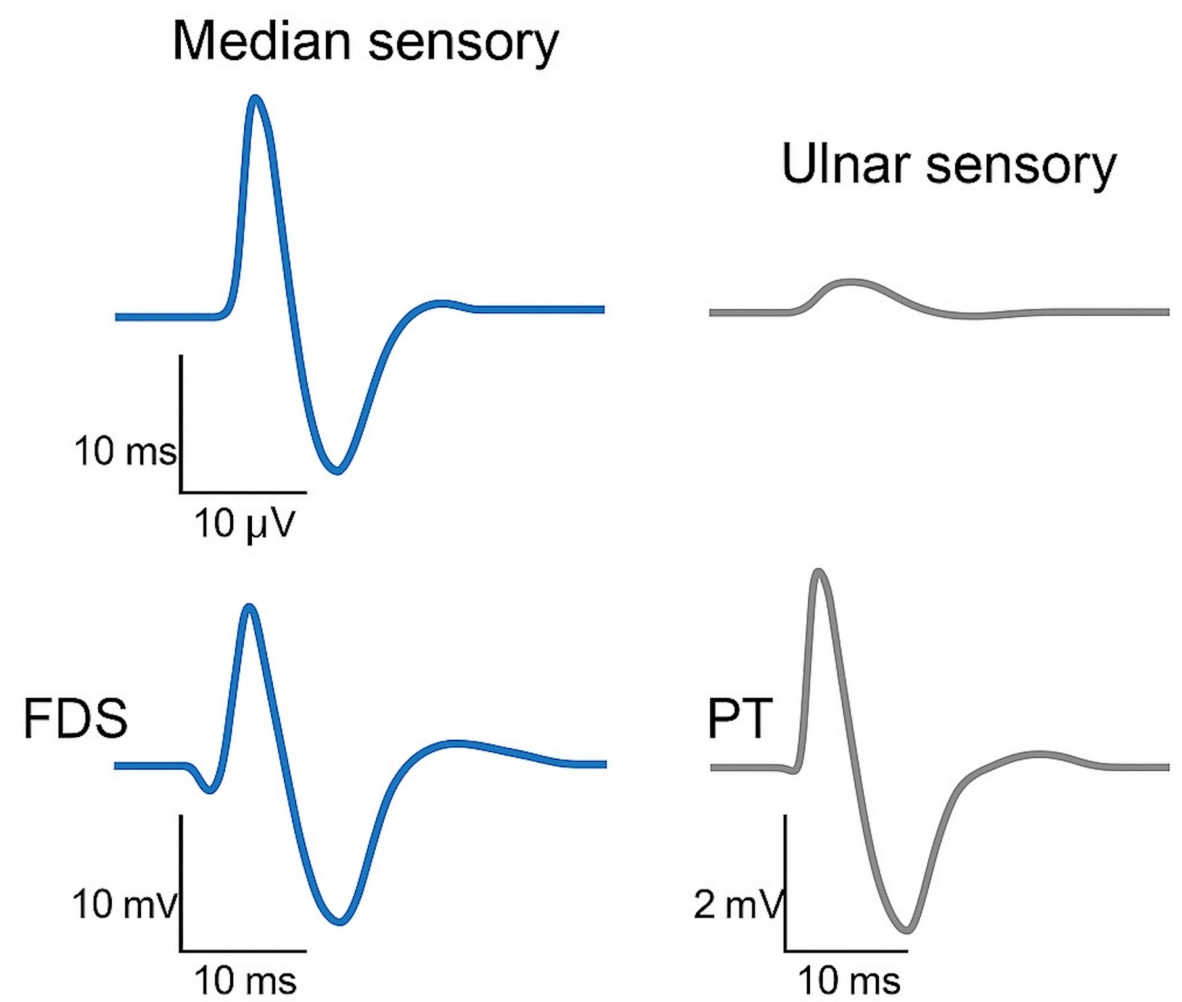
Representative Electromyography and Nerve Conduction Tracing FDS, flexor digitorum superficialis; PT, pronator teres.

Examination demonstrated sensory loss in the median distribution and impaired fine motor tasks. Electrodiagnostic testing (standard nerve conduction studies and needle electromyography) demonstrated a chronic focal median mononeuropathy localizing to the proximal forearm, consistent with axonal loss with demyelinating features. Background length-dependent sensory polyneuropathy was also present. Findings supported the clinical diagnosis and excluded radiculopathy or plexopathy (Figure 1).

Given persistent pain and functional limitation despite conservative therapy, the patient elected to trial percutaneous 60-day PNS lead (SPRINT^®^, SPR Therapeutics, Cleveland, OH) placement.

Intervention

Under sterile conditions, the proximal forearm to the elbow was prepared. Using ultrasound, the brachial artery and median nerve were identified, and a local anesthetic was administered to the skin and subcutaneous tissue overlying the target structures but outside the planned lead trajectory to avoid confounding stimulation testing. A 17-gauge insulated stimulating introducer was advanced in-plane to the median nerve (proximal to the cubital fossa), and test stimulation reproduced the patient’s typical paresthesia in the first three digits at comfortable amplitudes. A percutaneous 60-day PNS lead was deployed, then retested to confirm appropriate distribution. Post-procedure programming optimized frequency and pulse width, and the patient was instructed on device care and remote use.

Outcomes

At two-month follow-up, the patient reported greater than 50% reduction in pain intensity (average daily pain score recorded in pain diary down to 3/10 by numeric rating scale (NRS) at follow-up versus 8/10 pre-procedure) and improved dexterity enabling fuller participation in therapy. Continuous low-level stimulation was well tolerated. The PNS leads were removed at this time. The patient continued venlafaxine and capsaicin 0.1% cream, with plans for tapering based on functional progress. At one-year follow-up, the patient reported sustained 50% reduction in pain intensity (average daily pain score of 4/10 by NRS in pain diary) and had stopped using the capsaicin 0.1% cream. 

## Discussion

Median nerve injury after brachial arterial instrumentation is rare but clinically important. Broader reviews of arterial interventions confirm that nerve injuries, while uncommon, are most frequently reported with axillary and brachial access because of tight neurovascular relationships and challenges in achieving hemostasis; hematoma and pseudoaneurysm formation are frequent contributors [2]. Case reports also describe median neuropathy after brachial or axillary catheterization due to pseudoaneurysm compression, direct trauma, or ischemia [1,3-5].

Unlike many previously reported cases, which typically manifest within days to weeks of vascular access, our patient developed symptoms more than a year after a prolonged prone spine procedure with a brachial arterial line in place. The delayed presentation distinguishes this case and suggests a possible mechanism of chronic low-grade compression or microvascular ischemia rather than acute trauma. Electrodiagnostic findings showing both axonal loss and demyelinating features support a mixed injury pattern, differing from the primarily demyelinating lesions described in acute compressive neuropathies [3,4].

Few reports have detailed management strategies once such chronic focal median mononeuropathies develop. Conservative measures, including neuropathic medications, splinting, and physical therapy, often provide incomplete relief. Surgical exploration or decompression may be considered when a structural lesion such as a pseudoaneurysm or fibrotic entrapment is identified, but outcomes are variable and frequently incomplete [5-7].

In contrast, ultrasound-guided PNS has emerged as a minimally invasive, motor-sparing option for focal neuropathic pain refractory to conservative care. Published experiences demonstrate meaningful and durable analgesia in upper-extremity neuropathic conditions, including brachial plexus, suprascapular, and median nerve distributions [8-10]. Compared with spinal cord stimulation or repeated local anesthetic injections, PNS enables focal targeting, preserves motor function, and avoids the need for permanent implants. In our case, a 60-day percutaneous system provided more than 50% pain reduction and functional improvement, allowing the patient to resume activities of daily living.

This case adds to the limited literature describing post-arterial-access median neuropathies and highlights that even delayed presentations can respond to PNS. Awareness of this potential iatrogenic etiology is important for clinicians evaluating otherwise unexplained median neuropathic pain, especially when there is a history of brachial or axillary vascular access.

## Conclusions

Median nerve neuropathy can occur as a delayed complication of brachial arterial cannulation, even when the access event occurred months or years earlier. Clinicians should specifically ask about prior upper-extremity vascular lines and pursue focused electrodiagnostic testing when symptoms localize to the proximal median nerve.

For patients with persistent, medication-refractory focal neuropathic pain and no surgically correctable lesion, ultrasound-guided percutaneous PNS offers a practical, motor-sparing therapeutic option that can restore function and quality of life. Early recognition of this rare iatrogenic entity may facilitate timely referral for neuromodulation and improved outcomes.
